# Metabolic Responses to *Orientia tsutsugamushi* Infection in a Mouse Model

**DOI:** 10.1371/journal.pntd.0003427

**Published:** 2015-01-08

**Authors:** Jeeyoun Jung, Youngae Jung, Byoungchul Gill, Changhun Kim, Kyu-Jam Hwang, Young-Ran Ju, Hye-Ja Lee, Hyuk Chu, Geum-Sook Hwang

**Affiliations:** 1 Integrated Metabolomics Research Group, Western Seoul Center, Korea Basic Science Institute, Seoul, Republic of Korea; 2 KM Health Technology Research Group of Medical Research Division, Korea Institute of Oriental Medicine, Daejeon, Republic of Korea; 3 Division of Zoonoses, Center for Immunology and Pathology, Korea National Institute of Health, Chungcheongbuk-do, Republic of Korea; 4 Division of Metabolic Diseases, Center for Biomedical Sciences, Korea National Institute of Health, Chungcheongbuk-do, Republic of Korea; 5 Division of Influenza Virus, Center for Infectious Disease, Korea National Institute of Health, Chungcheongbuk-do, Republic of Korea; 6 Graduate School of Analytical Science and Technology, Chungnam National University, Daejeon, Republic of Korea; University of California San Diego School of Medicine, United States of America

## Abstract

Tsutsugamushi disease is an infectious disease transmitted to humans through the bite of the *Orientia tsutsugamushi*-infected chigger mite; however, host-pathogen interactions and the precise mechanisms of damage in *O. tsutsugamushi* infections have not been fully elucidated. Here, we analyzed the global metabolic effects of *O. tsutsugamushi* infection on the host using ^1^H-NMR and UPLC-Q-TOF mass spectroscopy coupled with multivariate statistical analysis. In addition, the effect of *O. tsutsugamushi* infection on metabolite concentrations over time was analyzed by two-way ANOVAs. Orthogonal partial least squares-discriminant analysis (OPLS-DA) showed distinct metabolic patterns between control and *O. tsutsugamushi*-infected mice in liver, spleen, and serum samples. *O. tsutsugamushi* infection caused decreased energy production and deficiencies in both remethylation sources and glutathione. In addition, *O. tsutsugamushi* infection accelerated uncommon energy production pathways (i.e., excess fatty acid and protein oxidation) in host body. Infection resulted in an enlarged spleen with distinct phospholipid and amino acid characteristics. This study suggests that metabolite profiling of multiple organ tissues and serum could provide insight into global metabolic changes and mechanisms of pathology in *O. tsutsugamushi*-infected hosts.

## Author Summary

Scrub typhus is an acute febrile illness caused by attacks of *Orientia tsutsugamushi*-carrying mites, and is the most prevalent febrile illness in the Asia-Pacific region. If not properly treated with antibiotics, patients often develop severe vasculitis that affects multiple organs, and the mortality rate can reach 30%. To explore the pathogenic mechanisms underlying the host-pathogen interaction, we characterized metabolic changes in various organs and the serum of *O. tsutsugamushi*-infected hosts. After *O. tsutsugamushi* infection, the host experienced decreased energy production, as well as a severe deficiency in re-methylation sources and glutathione, which impaired purine synthesis, DNA and protein methylation. In addition, abnormal pathways for phosphatidylcholine (PC) biosynthesis and phosphoethanolamine methylation were utilized in the enlarged spleen of *O. tsutsugamushi*-infected hosts. These results suggested that metabolic profiling could provide insight into global metabolic changes in *O. tsutsugamushi*-infected hosts, and increase our understanding of the pathogenic mechanisms of *O. tsutsugamushi*, as well as providing novel therapeutic targets for scrub typhus.

## Introduction

*Orientia tsutsugamushi*, a mite-borne disease, is the causative agent of scrub typhus (tsutsugamushi disease), the most prevalent febrile illness in the Asia-Pacific region [1], [2]. This disease is characterized by fever, exanthematous rash, eschar, pneumonitis, meningitis, myalgia, and diffuse lymphadenopathy, symptoms similar to those of other acute febrile illnesses such as murine typhus, dengue fever, and viral hemorrhagic fevers. Delayed or inappropriate treatment can lead to severe multi-organ failure [3], [4].

The pathological characteristics of *O. tsutsugamushi-*infection have been described in the literature [5]–[10]. A previous study [8] reported the development of an acute febrile illness within 8–10 days of bites from the larva of trombiculid mites (which carry *O. tsutsugamushi* in their salivary glands), with bacteremia present 1–3 days before the onset of fever. When mice were infected intraperitoneally, *O. tsutsugamushi* was observed in smears of peritoneal exudates, liver, spleen, kidney and lung, and splenomegaly and peritonitis were evident [5], [9], [10]. The extent of pathological change in host organs has been well documented by several studies, but the precise mechanism of damage caused by *O. tsutsugamushi* infection remains unclear. Additionally, the host-pathogen interaction has not been clearly defined. Thus, novel approaches are required to explore the pathogenesis of *O. tsutsugamushi*.

Metabolomics is the study of systemic biochemical profiles by analysis of biofluids and tissues. Recent metabolomic investigations have sought to elucidate the nature of host-parasite interactions. Several studies have examined the effects of infection by *Mycobacterium tuberculosis*, *Plasmodium falciparum* and *Salmonella* using various analytic platforms [11]–[14], revealing the systemic effects of parasite diversion of nutrients on host metabolism.

In particular, the application of several analytical platforms to complement the strengths and weaknesses of different types of equipment has been emphasized. For example, ^1^H NMR demonstrates high reproducibility and provides detailed information regarding the quantity and identity of metabolites, but shows low sensitivity and is not suitable for lipid profiling. Conversely, ultra-performance liquid chromatography-mass spectrometry (UPLC-MS) is highly sensitive and has a high capacity for analysis of diverse chemical characteristics, including lipid species [15], [16]. Thus, many metabolomic studies utilized NMR and LC-MS in a complementary fashion [17], [18].

In the present study, we used ^1^H-NMR and UPLC-Q-TOF mass spectroscopy-based metabolic profiling to characterize the responses of BALB/c mice to *O. tsutsugamushi* Karp infection. The metabolic changes in various organs and serum for sham control and *O. tsutsugamushi*-infected mice were also investigated to explore host-pathogen interactions in an *O. tsutsugamushi*-infected host system.

## Materials and Methods

### Cell Culture and Bacteria

L929, a mouse fibroblast cell line, was obtained from the American Type Culture Collection (Rockville, MD.). L929 was cultured in Dulbecco's modified Eagle's medium (DMEM; Gibco BRL) supplemented with 5% fetal bovine serum (Gibco BRL), 5 mM L-glutamine, penicillin (100 U/ml), and streptomycin (100 µg/ml) in a humidified atmosphere containing 5% CO_2_. The Karp strain of *O. tsutsugamushi* was propagated in monolayers of L929 cells, as described previously with slight modifications [19]. When more than 90% of the cells were infected, as determined by an indirect immunofluorescence antibody assay (IFA) technique [2], cells were collected, homogenized using a glass Dounce homogenizer (Wheaton, Inc.), and centrifuged at 500×*g* for 5 min.

The supernatants were stored in liquid nitrogen until use. The titers of inocula were determined as follows: the bacterial stock was serially diluted and inoculated onto L929 cell layers in a 24-well tissue culture plate containing 12-mm diameter glass cover slip. After the cells were infected with *O. tsutsugamushi* for 4 h in a humidified 5% CO_2_ atmosphere at 34°C, the culture medium was removed. The cells were washed with phosphate-buffered saline (PBS), fixed in 100% acetone for 10 min at −20°C, and stained by IFA. The number of infected-cell-counting units (ICU) of *O. tsutsugamushi* was determined by fluorescence microscopy [20].

### Animals

Six-week-old female BALB/c inbred mice (ORIENT BIO), weighing 17 to 18 g, were used throughout the study. The test mice were inoculated intraperitoneally (i.p.) with 3.5×10^4^ ICU of *O. tsutsugamushi* Karp in 200 µl of PBS. After inoculation, mice were randomly separated into four groups of 12 and monitored for seven days (one control mouse dropped out during the experiment). The mice were observed at least twice daily for mortality, morbidity, and body weight. To identify changes in metabolite levels in the liver, spleen and blood, mice were sacrificed at 4 and 7 days post-infection. After measuring the length and weight of the spleen and liver, respectively, they were stored in liquid nitrogen until use. Blood samples were collected into a plain blood correction tube which does not contain any anticoagulant. Then, immediately after separation from blood by centrifugation, serum samples were stored in liquid nitrogen until use.

The mice were housed in animal biosafety level three facilities, where they received water and food *ad libitum*. Approval for animal experiments was obtained from the institutional animal welfare committee (KCDC-023-11).

### NMR Sample Preparation and NMR Spectroscopy for Metabolic Profiling

Liver and spleen tissues were rapidly frozen in liquid nitrogen and stored at −80°C prior to NMR analysis. For metabolite extraction, each tissue sample (100 mg of spleen or 200 mg of liver) was placed into a 1.5-mL tube containing 2.8-mm zirconium oxide beads and homogenized twice at 5000 rpm with 350 µL of methanol (*d*_4_) and 150 µL of 0.2 M (pH 7.0±0.1) sodium phosphate buffer for 20 s using a Precellys 24 tissue grinder (Bertin Technologies, Ampère Montigny-le-Bretonneux, France). After homogenization, 210 µL of methanol-*d*_4_, 90 µL of 0.2 M (pH 7.0±0.1) sodium phosphate buffer and 400 µL of chloroform were added to the tube. The mixture was then vortexed vigorously for 1 min and allowed to separate for 15 min. Samples were centrifuged at 13000 rpm for 10 min at 4°C. The upper layer was transferred as 630-µL aliquots to new 1.5 mL Eppendorf tubes and mixed with 70 µL of 2.5 mM TSP (trimethylsilyl propionate) dissolved in D_2_O. The mixture was then centrifuged at 13000 rpm for 5 min. Supernatants (600 µL) were transferred to 5-mm NMR tubes.

Serum samples were collected and stored at −80°C until NMR analysis. Prior to NMR, frozen serum samples were thawed at room temperature and vortexed. Samples (100 µL serum with 200 µL saline) were transferred into 5-mm NMR outer tubes and 260 µL of 0.5 mM DSS [3-(trimethylsilyl)-1-propanesulfonic acid sodium salt] dissolved in D_2_O were transferred into the inner tube [21]. Three serum samples were not used for NMR analysis due to insufficient sample volume.

^1^H NMR spectra at 298 K were acquired using a VNMRS-600 MHz NMR spectrometer (Agilent Technologies Inc., Santa Clara, CA) with a triple-resonance HCN salt-tolerant cold probe. For spleen and liver tissue samples, a one-dimensional (1D) NOESYPRESAT pulse sequence was applied to suppress the residual water signal. The spectrum was collected with 64 transients into 33,784 data points using a spectral width of 8445.9 Hz, relaxation delay of 2.0 s, an acquisition time of 4.0 s, and a mixing time of 100 ms. For serum samples, the water-suppressed Carr–Purcell–Meiboom–Gill (CPMG) spin-echo pulse sequence (RD-90°- [τ-180°-τ] n-ACQ) was used to attenuate broad signals from proteins and lipoproteins. The ^1^H-NMR spectrum was collected with 128 transients into 32 K data points using a spectral width of 12019.2 Hz with a relaxation delay of 2.0 s and an acquisition time of 2.662 s. The individual CPMG spin echo (in total 0.8 ms) was repeated 80 times, resulting in a total spin-spin relaxation delay of 64 ms.

### Data Processing and Multivariate Statistical Analysis

All NMR spectra were phased, baseline corrected and divided into 0.005 ppm bins using Chenomx NMR suite 7.1 (Chenomx, Edmonton, AB, Canada) software. The binning data were normalized with the total area of each spectrum by excluding the water resonance (4.19–5.21, 4.88–4.67 and 4.88–6.67 ppm for serum, liver and spleen, respectively). Binning data files were imported into MATLAB (R2008a, Mathworks, Inc., 2008), and all spectra were aligned using the correlation-optimized warping method, which shifted, stretched, and shrunk the segmented spectra to maximize the correlation coefficient between the segments of a spectrum and target spectrum (user-defined segmentation), resulted in the good alignment in single peaks as well as broad misalignment areas with minimizing the loss of resolution [22]. The resulting data sets were imported into SIMCA-P version 12.0 (Umetrics, Umeå, Sweden) for multivariate analysis, and Pareto-scaled [23]. Orthogonal partial least squares- discriminant analysis (OPLS-DA) was conducted, and the quality of each model was determined based on the goodness of fit parameter (*R^2^*), and goodness of prediction parameter (*Q^2^*) [24]. In addition, reliability of each OPLS-DA model was validated by CV-ANOVA test [25].

NMR spectral data analysis was accomplished using targeted profiling with Chenomx NMR Suite 7.1, and concentrations were determined using the 600 MHz library from Chenomx NMR Suite 7.1, which compares the integral of a known reference signal (DSS, TSP) with signals derived from a library of compounds containing chemical shifts and peak multiplicities. The ambiguous peaks caused by overlap or slight shifts were confirmed by spiking samples with standard compounds and utilized two-dimensional (2D) total correlation spectroscopy (TOCSY) and correlation spectroscopy (COSY) experiments for liver and spleen samples (S1 Fig.).

### Analysis of Spleen Lipid Metabolites

Spleen lipid metabolites were extracted with a chloroform: methanol mixture (2∶1, v/v), as described by Folch et al. [26]. Lipid extracts were diluted with an isopropanol: acetonitrile: water mixture (2∶1∶1, v/v/v) and 10 µl was used for LC/MS analysis. LC-MS analysis was performed on a triple TOF™ 5600 MS/MS system (AB SCIEX, Concord, Canada) combined with an Ultra-performance Liquid Chromatography system (Waters, Milford, MA). Separations were accomplished on an Acquity UPLC HSS C18 column (2.1×100 mm) with 1.8-µm particles (Waters, Milford, MA). A binary gradient system consisting of acetonitrile and water (4∶6, v/v) with 10 mM ammonium acetate was used as eluent A. Eluent B consisted of acetonitrile and isopropanol (1∶9, v/v) with 10 mM ammonium acetate. The gradient profile was 40% B at 0–3.5 min, 70% B at 8.5 min, 75% B at 12 min, 99% B at 12–13 min, and 40% B at 13.1–15 min. The flow rate was kept at 0.35 mL/min for 15 min. The mass spectrometer was operated in electrospray ionization (ESI) positive and negative modes, and the mass range was set at m/z 100–1500. The following parameter settings were used: ion spray voltage of 5500 V, temperature of 500°C, curtain gas of 30 psi, declustering potential of 90 V, and collision energy of 10 V. In addition, information-dependent acquisition (IDA) was used to trigger acquisition of MS/MS spectra for ions matching the IDA criteria. MS/MS experiments were performed with collision energies of 40 V and collision energy spreads of 15 V. Mass accuracy was maintained by the automated calibrant delivery system (AB Sciex, Concord, Canada) interfaced to the second inlet of the DuoSpray source. Experiments were performed in duplicate, and percent relative standard deviation (%RSD) for duplicate analysis of homogenized samples was used to measure precision.

All MS data, including retention times, m/z, and ion intensities, were extracted using the Markerview software (AB SCIEX, Concord, Canada) incorporated in the instrument, and the resulting MS data were assembled into a matrix. Ion intensity was normalized with the internal standard (1-heptadecanoyl-2-myristoleoyl-sn-glycero-3-phosphocholine), and the mean of duplicate results, showing <10% RSD, was utilized in statistical analysis. Metabolites were identified using the lipidmap (www.lipidmaps.org) and human metabolome (www.hmdb.ca) databases, and confirmed using standard compounds based on both retention times and mass spectra.

### Enzyme Activity Assays

Activities of hydroxymethylglutaryl CoA reductase (HMG-CoAR) and 3-hydroxybutyrate dehydrogenase were measured using ELISA kits for mouse HMG–CoAR and mouse 3-hydroxybutyrate dehydrogenase (BDH2) (Cusabio Biotech Co. Ltd, Wuhan, China), respectively, according to the manufacturer's instructions, and enzyme activities were measured in duplicate.

### Statistical Analysis

A two-way ANOVA combined with Bonferroni posttests was performed using GraphPad PRISM version 5.0 (GraphPad Software, Inc., La Jolla, CA) to analyze the influence of time and infection on metabolite concentrations, body weight, and spleen length. All enzyme activities including AST, ALT, HMG–CoA reductase and 3-hydroxybutyrate dehydrogenase were subjected to Mann-Whitney U test using SPSS 15.0 for Windows (SPSS, Chicago, IL). Statistical significance was set at *P*<0.05.

## Results

### Body Weight, Spleen Length and Liver Function

**Figure 1:**
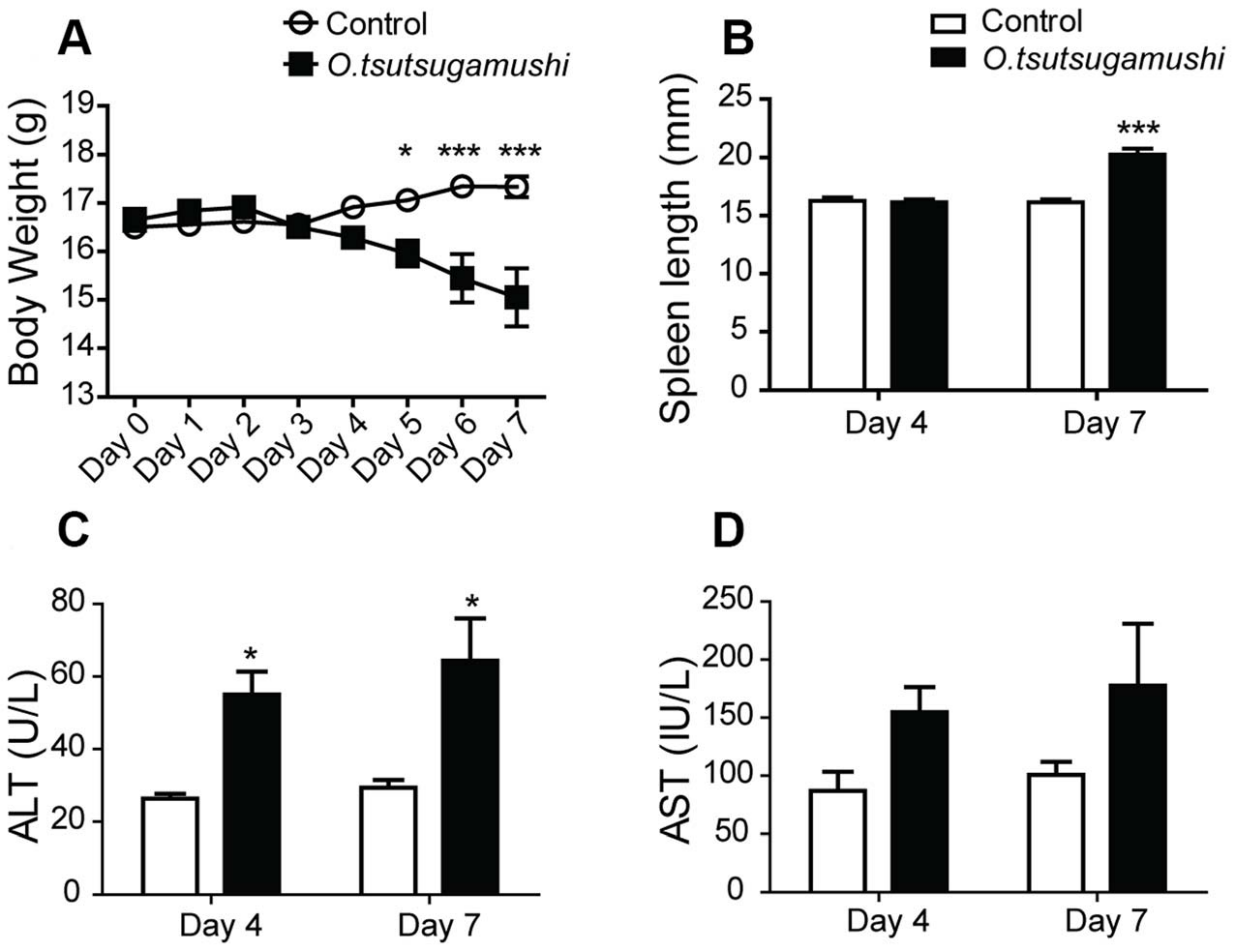
Body weight, spleen length, and AST and ALT levels. *, *p*<0.05; ** *p*<0.01; ***, *p*<0.001 for control vs. *O. tsutsugamushi*-infected mice.

By day 3 after inoculation, several clinical signs of infection such as ruffled hair, slowed movement, and body weight changes were evident in *O. tsutsugamushi*-infected mice. Compared with control mice, the body weights of *O. tsutsugamushi*-infected mice decreased by 3.7% and 13.2% on days 4 and 7 after infection, respectively (Fig. 1a), and the spleens of infected mice were significantly longer than those of control mice on day 7 (Fig. 1b). Levels of ALT, an indicator of liver dysfunction, also increased after infection (Fig. 1c and d).

### ^1^H NMR Spectroscopy and Pattern-Recognition Analysis

Representative 600-MHz ^1^H-NMR spectra of liver, spleen, and serum samples obtained from control and *O. tsutsugamushi*-infected mice are shown in S2-S4 Figs. We used these spectra to identify endogenous metabolites, and 19, 17 and 15 metabolites in liver, spleen and serum, respectively, were identified by 2D TOCSY spectra and/or spiking experiments (Tables 1–3).

**Table 1:** Summary of metabolites in liver tissue.

Metabolites	Chemical shift (multiplicity); Identification method	↑/↓^a^	*p*_infection_	*p*_time_	*p*_interaction_
Isoleucine	0.95 (t), 1.01 (d); Spiking, TOCSY	↓	<0.001*	0.010*	0.380
Leucine	0.97 (dd), 1.73(m); Spiking, TOCSY	↓	<0.001*	0.485	0.060
Valine	1.00 (d), 1.05 (d), 2.28 (m); Spiking, TOCSY	↓	<0.001*	0.383	0.416
Isopropanol	1.16 (d), 3.98(m); Spiking, TOCSY	- b	0.141	0.337	0.266
3-Hydroxybutyrate	1.18 (d), 2.31 (m), 2.35 (m), 4.11(m); Spiking, TOCSY	↑	<0.001*	0.735	0.483
Lactate	1.32 (d), 4.04 (q); TOCSY	↓	<0.001*	0.891	0.569
Alanine	1.48 (d), 3.72(q); TOCSY	↓	<0.001*	0.059	0.946
Acetate	1.90 (s); Spiking	-	0.156	0.010*	0.615
Glutamate	2.03 (m), 2.14 (m), 2.37(m), 3.69(dd); Spiking, TOCSY	↓	0.028*	0.015*	0.405
Glutathoine	2.15 (m), 2.54 (m), 2.90-2.98 (m), 3.74(m); Spiking, TOCSY	↓	<0.001*	0.943	0.017*
Creatine	3.03 (s), 3.92(s); Spiking	-	0.765	0.139	0.061
Glucose	3.18 (dd), 3.35–3.46 (m), 3.67–3.74 (m), 3.80 (m), 3.87 (dd), 4.57 (d), 5.18 (d); TOCSY	↓	<0.001*	0.613	0.450
Choline	3.21 (s), 3.51 (m), 4.05 (m); Spiking	↓	<0.001*	<0.001*	0.090
O-Phosphocholine	3.22 (s), 3.58 (m), 4.18 (m); Spiking	↑	<0.001*	0.030*	0.009*
Betaine	3.27 (s), 3.89 (s); Spiking	↓	<0.001*	<0.001*	0.010*
Glycine	3.53 (s); Spiking	↓	<0.001*	0.019*	0.781
Glycerol	3.54 (dd), 3.62 (dd), 3.77(m); Spiking	↓	<0.001*	0.899	0.719
Fumarate	6.52 (s); Spiking	-	0.382	0.155	0.182
Nicotinurate	7.60 (dd), 8.27 (m), 8.71 (dd), 8.96 (d); TOCSY	↓	<0.001*	0.066	0.546

a Arrows (↓ and ↑) represent a decrease or increase in metabolite levels with significant changes in *O. tsutsugamushi*-infected mice compared with control mice on day 4 and/or day 7.

b No significant change. Levels were estimated based on intensities of ^1^H-NMR spectra of the liver following spectral normalization. s, singlet; d, doublet; q, quartet; t, triplet, m, multiplet; br, broad; *, *p*<0.05.

**Table 2:** Summary of metabolites in spleen tissue.

Metabolites	Chemical shift (multiplicity); Identification method	↑/↓^a^	*p*_infection_	*p*_time_	*p*_interaction_
Isoleucine	0.95 (t), 1.02 (d); Spiking, TOCSY	↑	<0.001*	0.273	0.117
Leucine	0.97 (dd), 1.73(m); Spiking, TOCSY	↑	<0.001*	0.997	0.233
Valine	1.00 (d), 1.05 (d), 2.26 (m); TOCSY	↑	0.001*	0.853	0.689
Isopropanol	1.16 (d), 3.98(m); Spiking, TOCSY	↑	0.001*	<0.001*	0.018*
3-Hydroxybutyrate	1.18 (d), 2.29(m), 2.36(m), 4.11(m); TOCSY	↑	<0.001*	0.759	0.270
Lactate	1.32 (d), 4.07 (q); TOCSY	↑	0.040*	0.116	0.361
Threonine	1.32 (d), 3.48 (d), 4.20 (m); Spiking, TOCSY	- b	0.104	0.876	0.033*
Alanine	1.47 (d), 3.71(q); TOCSY	-	0.077	0.620	0.617
Glutamate	2.03 (m), 2.14 (m), 2.37(m), 3.68 (dd); TOCSY	-	0.612	0.644	0.508
Taurine	3.18 (t), 3.36 (t); TOCSY	-	0.845	0.029*	0.021*
Choline	3.21 (s), 3.51 (m), 4.06 (m); Spiking	↑	0.014*	0.011*	0.001*
O-Phosphocholine	3.22 (s), 3.58 (m), 4.16 (m); Spiking	↓	<0.001*	0.184	0.002*
O-Phosphoethanolamine	3.20 (m), 3.99 (m); Spiking, TOCSY	↑	<0.001*	0.012*	0.011*
Betaine	3.27 (s), 3.88 (s); Spiking	↓	0.001*	<0.001*	0.022*
Myo-inositol	3.27 (t), 3.46 (dd), 3.61 (t), 4.04 (t); Spiking, TOCSY	-	0.13	0.057	0.097
Glycine	3.49 (s); Spiking	-	0.135	0.166	0.087
Formate	8.44 (s); Spiking	-	0.393	0.866	0.329

a Arrows (↓ and ↑) represent a decrease or increase in metabolite levels with significant changes in *O. tsutsugamushi*-infected mice compared with control mice on day 4 and/or day 7.

b No significant change. Levels were estimated based on intensities of ^1^H-NMR spectra of a spleen following spectral normalization. s, singlet; d, doublet; q, quartet; t, triplet, m, multiplet; br, broad; *, *p* <0.05.

**Table 3:** Summary of metabolites in serum.

Metabolites	Chemical shift (multiplicity); Identification method	↑/↓^a^	*p*_infection_	*p _t_*_ime_	*p*_interaction_
Isoleucine	0.93 (t), 0.98 (d); 1D	- b	0.126	0.191	0.810
Leucine	0.93 (d), 0.96 (d), 1.70 (m); Spiking	-	0.510	0.028*	0.082
Valine	0.96 (d), 1.02 (d), 2.27 (m); 1D	-	0.133	0.317	0.349
3-Hydroxybutyrate	1.17 (s), 2.28 (dd), 2.38 (dd), 4.12 (dd); 1D	↑	<0.001*	0.759	0.412
Lactate	1.30 (d), 4.09 (q); 1D	↓	0.011*	0.200	0.780
Alanine	1.45 (d); 1D	-	0.081	0.014*	0.233
Acetate	1.91 (s); Spiking	-	0.211	0.952	0.387
Glutamate	2.00 (m), 2.12 (m), 2.22 (m); 1D	-	0.501	0.600	0.913
Pyruvate	2.34 (s); Spiking	↓	0.018*	0.303	0.034*
Citrate	2.50 (d), 2.66 (d); Spiking	↓	0.003*	0.800	0.016*
Creatine	3.01 (s), 3.92 (s); Spiking	↑	0.037*	0.248	0.025*
Choline	3.18 (s), 3.51 (m), 4.06 (m); Spiking	-	0.083	0.172	0.991
Glucose	3.20 (t), 3.35-3.45 (m), 3.51 (dd), 3.67-3.83 (m), 3.87 (dd), 4.62 (d), 5.21 (d); 1D	↓	<0.001*	0.841	0.592
Betaine	3.23 (s), 3.90 (s); Spiking	↓	0.005*	0.564	0.339
Glycerol	3.53 (dd), 3.63 (dd); 1D	-	0.270	0.106	0.306

a Arrows (↓ and ↑) represent a decrease or increase in metabolite levels with significant changes in *O. tsutsugamushi*-infected mice compared with control mice on day 4 and/or day 7.

b No significant change. Levels were estimated based on intensities of ^1^H-NMR spectra of serum following spectral normalization. s, singlet; d, doublet; q, quartet; t, triplet, m, multiplet; br, broad; *, *p*<0.05.

**Figure 2:**
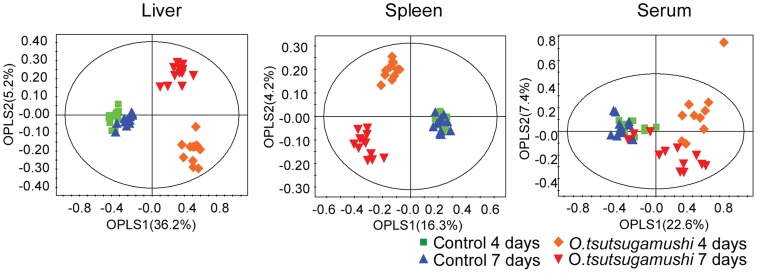
OPLS-DA score plots derived from NMR spectra of the liver, spleen, and serum. **Number of mice**: for liver and spleen model, Control 4 days (n = 12), Control 7 days (n = 12), *O. tsutsugamushi* 4 days (n = 11) *O. tsutsugamushi* 7 days (n = 12); for serum model, Control 4 days (n = 10), Control 7 days (n = 12), *O. tsutsugamushi* 4 days (n = 10) *O. tsutsugamushi* 7 days (n = 12).

To identify differences in the metabolite levels between control and *O. tsutsugamushi*-infected mice, orthogonal partial least squares- discriminant analysis (OPLS-DA) was applied to the NMR spectra of the liver, spleen, and serum samples. OPLS-DA score plots (Fig. 2) showed clear differences in the liver, spleen, and serum samples of control and infected mice, indicating significant changes in the metabolism of *O. tsutsugamushi*-infected mice. *R^2^Y* and *Q^2^Y* values of the OPLS-DA models for the liver, spleen, and serum samples were 0.885 and 0.624, 0.900 and 0.486, and 0.630 and 0.331, respectively. In addition, all OPLS-DA models had *p* values lower than 0.05 (*p*<0.001 [liver], *p*<0.001 [spleen], and *p* = 0.02 [serum]) in CV-ANOVA test.

*O. tsutsugamushi*-infected groups also showed significant differences in metabolite levels between days 4 and 7, but a change in control mice was not apparent, indicating that progression of *O. tsutsugamushi* infection had a marked effect on the metabolism of mice compared to the time-dependent metabolic changes that occur during natural aging.

### Effects of *O. tsutsugamushi* Infection on Liver Metabolite Levels

Two-way ANOVAs were applied to investigate the effect of *O. tsutsugamushi* infection and time on metabolite concentrations in liver tissues. Among the 19 metabolites detected in liver tissues, levels of 15 metabolites (isoleucine, leucine, valine, 3-hydroxybutyrate, lactate, alanine, glutamate, glutathione, glucose, choline, O-phosphocholine, betaine, glycine, glycerol and nicotinurate) differed significantly between control and *O. tsutsugamushi*-infected mice. Furthermore, betaine, glutathione, and O-phosphocholine showed an interaction (infection × time) effect. We found that isoleucine, leucine, valine, lactate, alanine, glutamate, glutathione, glucose, choline, O-phosphocholine, betaine, glycine, glycerol, and nicotinurate levels decreased, while 3-hydroxybutyrate and O-phosphocholine levels increased. The significant decrease in the majority of metabolite levels indicates that *O. tsutsugamushi* infection affected the liver (Table 1), and all significantly altered metabolites except nicotinurate also showed high values of weight in OPLS-DA model (w>|0.04|, S5 Fig.).

**Figure 3:**
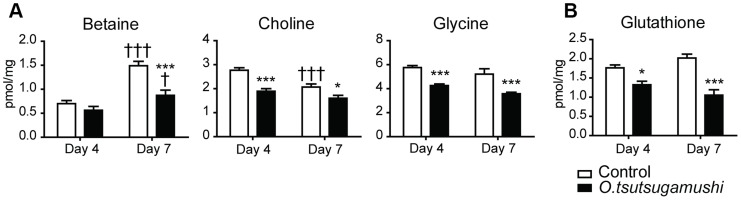
Alteration of levels of metabolites involved in methylation pathways, including betaine, choline and glycine (a) and glutathione (b) in the liver. Bars represent averages of duplicates ± SEM.; *, *p*<0.05; **, *p*<0.01; ***, *p*<0.001 for the control vs. *O. tsutsugamushi*-infected mice; †, *p*<0.05; ††, *p*<0.01; †††, *p*<0.001 of day 4 vs. 7.

All metabolites (choline, betaine and glycine) associated with remethylation cycles providing substrates for transmethylation were significantly decreased, and the antioxidant metabolite (glutathione) was also decreased on days 4 and 7 post-infection (Fig. 3).

### Effects of *O. tsutsugamushi* Infection on Spleen Metabolite Levels

In the spleen, 10 metabolites (isoleucine, leucine, valine, isopropanol, 3-hydroxybutyrate, lactate, choline, O-phosphocholine, O-phosphoethanolamine and betaine) were affected by infection. Betaine, choline, isopropanol, O-phosphocholine, O-phosphoethanolamine, taurine and threonine showed interaction (infection × time) effects. We found that isoleucine, leucine, valine, isopropanol, 3-hydroxybutyrate, lactate, choline and O-phosphoethanolamine levels increased, while those of O-phosphocholine and betaine decreased after infection (Table 2), and all significantly altered metabolites also showed high values of weight in OPLS-DA model (w>|0.04|, S6 Fig.).

### Effects of *O. tsutsugamushi* Infection on Serum Metabolite Levels

In sera, 7 metabolites (3-hydroxybutyrate, lactate, pyruvate, citrate, creatine, glucose and betaine) were affected by infection. 3 metabolites (pyruvate, citrate and creatine) showed interaction (infection × time) effects. We found that levels of lactate, pyruvate, citrate, glucose and betaine decreased significantly post-infection, while those of 3-hydroxybutyrate and creatine increased (Table 3), and all significantly altered metabolites also showed high values of weight in multivariate analysis (w>|0.04|, S7 Fig.).

### Energy Source Utilization in *O. tsutsugamushi*-Infected Mice

**Figure 4:**
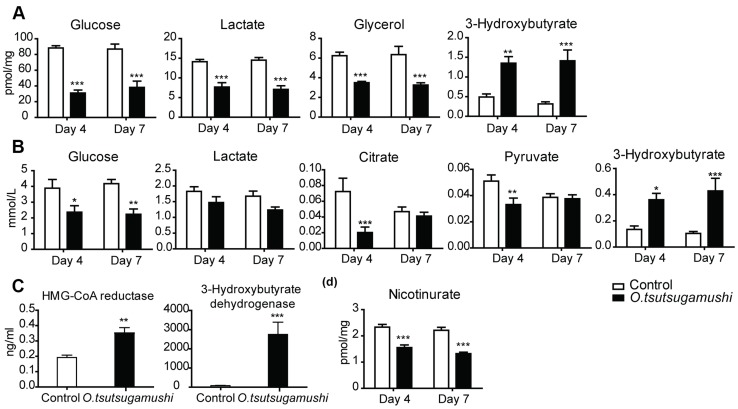
Changes in energy metabolism-related metabolites in liver (a) and serum (b) samples of *O. tsutsugamushi*-infected mice, activities of HMG–CoA reductase and 3-hydroxybutyrate dehydrogenase (c), and level of nicotinurate in livers of *O. tsutsugamushi*-infected mice (d). Bars represent averages of duplicates ± SEM.; *, *p*<0.05; **, *p*<0.01; ***, *p*<0.001 of control vs. *O. tsutsugamushi-*infected mice.

Interestingly, among the metabolites whose levels were decreased in the liver and/or serum of *O. tsutsugamushi*-infected mice, several metabolites (glucose, lactate, pyruvate, glycerol, and citrate) are associated with glycolysis and the TCA cycle. In addition, levels of ketone bodies and 3-hydroxybutyrate were significantly elevated in the liver and serum of infected mice (Fig. 4a and b). This indicates that energy source utilization was significantly affected by *O. tsutsugamushi* infection.

To identify the metabolic pathways affected by infection, we measured the activities of HMG–CoA reductase and 3-hydroxybutyrate dehydrogenase (Fig. 4c). In *O. tsutsugamushi*-infected mice, the activities of both enzymes were significantly increased, indication beta oxidation; i.e., breakdown of fatty acids to produce energy. Nicotinurate was also significantly decreased in the liver of *O. tsutsugamushi*-infected mice (Fig. 4d).

### Alternation of Lipid Metabolism in Enlarged Spleen of *O. tsutsugamushi*-Infected Mice

**Figure 5:**
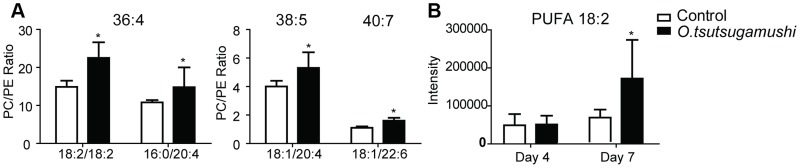
Ratio of phosphatidylcholine (PC) to phosphatidylethanolamine (PE) (a), and polyunsaturated fatty acids (PUFA) (b) in the enlarged spleens of *O. tsutsugamushi*-infected mice. Bars represent averages of duplicates ± SEM.; *, *p*<0.05 for the control vs. *O. tsutsugamushi*-infected mice.

In NMR data for the spleen tissues, we observed significant alterations of choline, O-phosphocholine, and O-phosphoethanolamin, which are involved in phosphatidylcholine (PC) and phosphatidylethanolamine (PE) metabolism. Thus, we further analyzed PC, PE and lipid metabolites using UPLC-MS. In *O. tsutsugamushi*-infected mice, long-chain fatty acid levels, PC and PE levels, PC/PE ratios, and polyunsaturated fatty acid levels in the spleen (18∶2) were all significantly increased when compared to control mice (Fig. 5, S1 and S2 Tables).

## Discussion

To explore host–*O. tsutsugamushi* interactions, we first applied NMR-based metabolic profiling to the liver, spleen and serum of *O. tsutsugamushi*-infected mice. At 5 days post-infection, we observed significantly reduced body weight, splenomegaly, and elevated levels of AST and ALT in *O. tsutsugamushi*-infected mice. PLS-DA analysis showed that *O. tsutsugamushi* infection particularly affected liver metabolism.

Significantly elevated ALT levels post-infection indicated hepatocyte destruction, leading to alteration of energy production by the host liver. Decreased levels of glycolysis and TCA cycle intermediate metabolites—such as glucose, lactate, pyruvate, glycerol and citrate—were also found in the serum and/or liver of *O. tsutsugamushi-*infected mice.

Previous studies have suggested that *O. tsutsugamushi* organisms obtain the majority of their energy from sources other than sugar catabolism, due to a lack of sugar-phosphate transporters or hexokinase [27], [28]. Consistent with this assumption, we found decreased levels of branched-chain-amino-acids (BCAA; isoleucine, leucine and valine) in the serum and liver of *O. tsutsugamushi-*infected mice. These changes were likely due to the higher energy efficiency of BCAA in conditions of liver dysfunction [29]. Min *et al*. stated that in *O. tsutsugamushi*, the TCA cycle begins with alpha-ketoglutarate, which is converted from glutamate likely obtained from the host cell. In this study, we found decreased glutamate levels in the livers of *O. tsutsugamushi*-infected mice.

We observed excess beta-oxidation of fatty acids as evidenced by elevated levels of 3-hydroxybutyrate, HMG–CoA reductase, and 3-hydroxybutyrate dehydrogenase in *O. tsutsugamushi*-infected hosts. These results indicate that *O. tsutsugamushi* infection affected the energy metabolism of host mice, suppressing oxidation of carbohydrates by reducing the availability of substrates for glycolysis and the TCA cycle, and increasing fatty acid oxidation for energy generation. Unlike other *Rickettsia*, *O. tsutsugamushi* has no beta-oxidation system for fatty acid energy generation [30]. Therefore, the ketone body, 3-hydroxybutyrate, is obtained from the host during *O. tsutsugamushi* infection, unlike other infectious diseases [31], and serves as a carbohydrate substitute during energy production.

Nicotinuric acid is the major product of nicotinic acid metabolism, and serves as a simple quantitative index of hepatic biotransformation of nicotinic acid [32]. Reduced levels of nicotinuric acid are indicative of inhibited synthesis of nicotinate metabolites, such as NADH, NAD, NAD+, and NADP, which play essential roles in energy metabolism and DNA repair [33].

In this study, we also observed decreased levels of all metabolites associated with folate-mediated one-carbon metabolism (choline, betaine and glycine) in the livers of *O. tsutsugamushi*-infected mice. Choline and betaine are particularly necessary for methionine synthesis in the homocysteine remethylation process; methionine subsequently provides methyl groups in transmethylation reactions [34]–[37]. Therefore, transmethylation in the liver is affected by *O. tsutsugamushi* infection, although *O. tsutsugamushi* contains only a subset of the genes necessary for folate metabolism [27]. Methylation defects found in HIV-infected patients are thought to be linked to neurological damage and immune cell depletion [38]–[40]. Some reports have found that *O. tsutsugamushi* infection involves the central nervous system and may cause Guillain–Barré syndrome, and meningitis [41]–[43], which may be attributed to the lack of methylation in *O. tsutsugamushi-*infected hosts reported that *O. tsutsugamushi* affected the host cell antioxidant system and free radical levels [44]. In agreement with this result, we found decreased levels of glutathione (which plays an important role in defense against oxidative stress) in the liver of *O. tsutsugamushi*-infected mice.

*O. tsutsugamushi* infection resulted in splenomegaly and alteration of splenic components. A previous TEM study of *O. tsutsugamushi-*infected spleens found large numbers of phagosomes and phagolysosomes in the intracytoplasm of macrophages, swollen and decrepit red blood cells, and the presence of additional membranes enveloping *O. tsutsugamushi* organisms. Thus, the high levels of PC and PE observed in our study may be due to phagocytic destruction of erythrocytes [45], [46]. We observed both increases and decreases in the levels of O-phosphocholine and O-phosphoethanolamine, PE and PC, long-chain fatty acids, and polyunsaturated fatty acids. These changes may be related to the breakdown of *O. tsutsugamushi-*enveloping membranes and/or spleen cell membranes in addition to breakdown of erythrocytes. In *O. tsutsugamushi-*infected mice, splenic enlargement may be related to increased BCAA levels and lymphocyte proliferation [47], [48]. However, further studies are needed to definitively determine the mechanisms of lipid and BCAA changes in the *O. tsutsugamushi-*infected spleen.

Global metabolite profiling of various organ tissues and serum provided insight into the effects of *O. tsutsugamushi* infection on host metabolism. *O. tsutsugamushi*-infected mice exhibited altered metabolism of the liver, as in energy source utilization, and the spleen. In conclusion, a global systems biology approach provides more information on host-pathogen interactions, and will likely facilitate efficient diagnosis and treatment of *O. tsutsugamushi*.

## Supporting Information

S1 Fig **Expansion of 2D TOCSY spectra of liver (a) and spleen (b) extracts.**
(TIF)

S2 Fig **Representative 600MHz ^1^H – NMR spectra of liver extract of control mice for day 4 (a) control mice for day 7 (b),** ***O. tsutsugamushi*** **-infected mice for day 4 (c),** ***O. tsutsugamushi*** **-infected mice for day 7 (d).** Key, 1. TSP, 2. Isoleucine, 3. Leucine, 4. Valine, 5. Isopropanol, 6. 3-Hydroxybutyrate, 7. Lactate, 8. Alanine, 9. Acetate, 10. Glutamate, 11. Glutathione 12. Creatine, 13. Glucose, 14. Choline, 15. O-Phosphocholine, 16. Betaine, 17. Glycine, 18. Glycerol, 19. Fumarate, 20. Nicotinurate.
(TIF)

S3 Fig **Representative 600 MHz ^1^H – NMR spectra of spleen extract of control mice for day 4 (a) control mice for day 7 (b),** ***O. tsutsugamushi*** **-infected mice for day 4 (c),** ***O. tsutsugamushi*** **-infected mice for day 7 (d).** Key, 1. TSP, 2. Isoleucine, 3. Leucine, 4. Valine, 5. Isopropanol, 6. 3-Hydroxybutyrate, 7. Lactate 8. Threonine, 9. Alanine, 10. Glutamate, 11. Taurine, 12.O-Phosphoethanolamine 13. Choline, 14. O-Phosphocholine, 15 Betaine, 16. Glycine, 17. Myo-inositol.
(TIF)

S4 Fig **Representative 600 MHz ^1^H – NMR spectra of serum of control mice for day 4 (a) control mice for day 7 (b),** ***O. tsutsugamushi*** **-infected mice for day 4 (c),** ***O. tsutsugamushi*** **-infected mice for day 7 (d).** Key, 1. DSS, 2. Isoleucine 3. Leucine, 4. Valine, 5. 3-Hydroxybutyrate, 6. Lactate, 7. Alanine, 8. Acetate, 9. Glutamate, 10. Pyruvate, 11. Citrate, 12. Creatine, 13. Choline, 14. Glucose, 15. Betaine, 16. Glycerol.
(TIF)

S5 Fig **Loading plots of OPLS-DA model derived from ^1^H NMR spectra of liver tissue: (a), full scale and (b), expanded scale.** Variables having high values of weight in OPLS-DA model were marked with red square.
(TIF)

S6 Fig **Loading plots of OPLS-DA model derived from ^1^H NMR spectra of spleen tissue: (a), full scale and (b), expanded scale.** Variables having high values of weight in OPLS-DA model were marked with red square.
(TIF)

S7 Fig **Loading plots of OPLS-DA model derived from ^1^H NMR spectra of serum: (a), full scale and (b, c), expanded scale.** Variables having high values of weight in OPLS-DA model were marked with red square.
(TIF)

S1 Table **Analysis of phosphatidylcholin and phosphatidylethanolamine of spleen.**
(DOCX)

S2 Table **Analysis of fatty acids of spleen.**
(DOCX)
