# Supplementary figures and images for: Metabolic Responses to Orientia tsutsugamushi Infection in a Mouse Model
Source: PLoS Negl Trop Dis. 2015 Jan 8;9(1):e3427. doi: 10.1371/journal.pntd.0003427 (PMC4287389; doi:10.1371/journal.pntd.0003427)

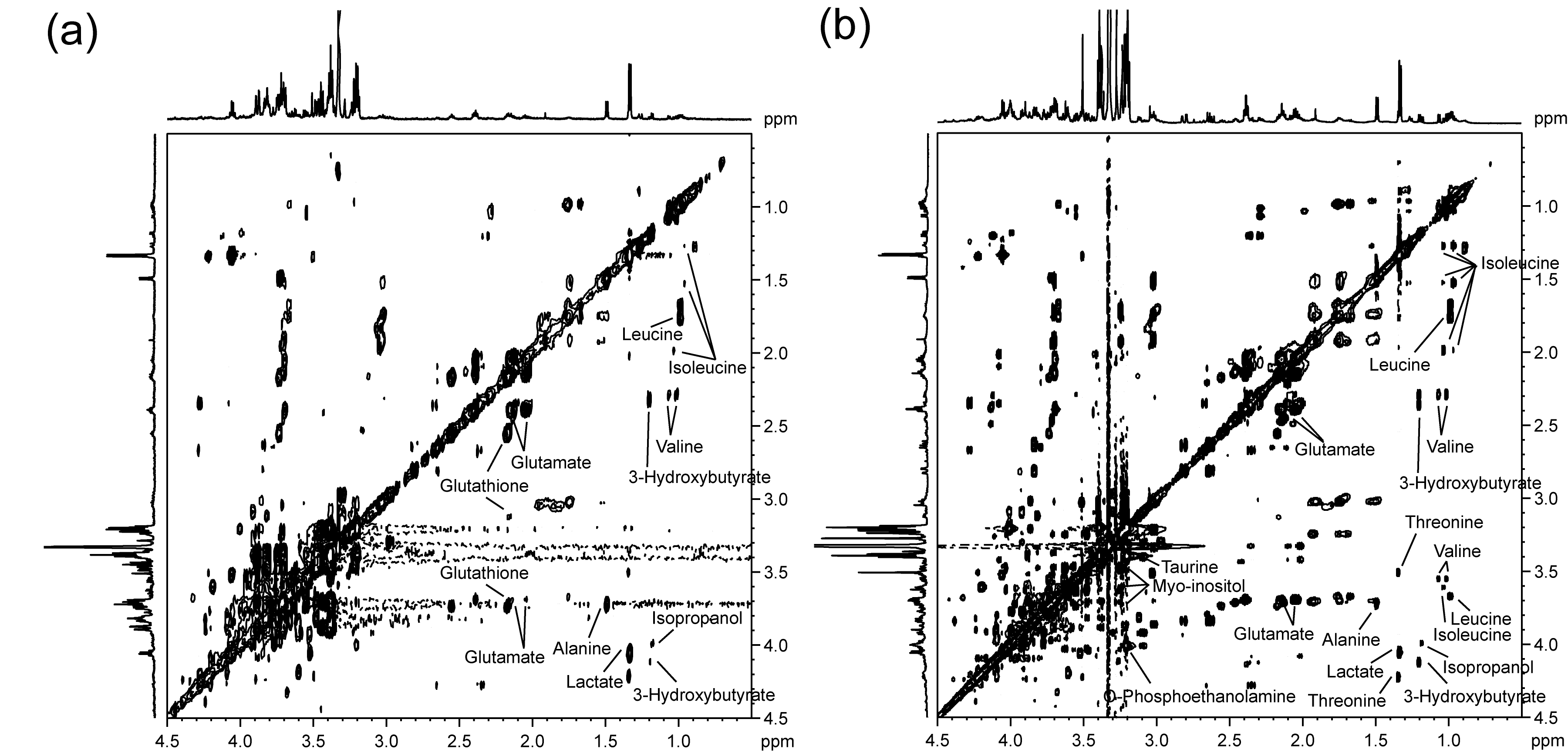

Supplement: S1 Fig — Expansion of 2D TOCSY spectra of liver (a) and spleen (b) extracts. (TIF) [file pntd.0003427.s001.tif]

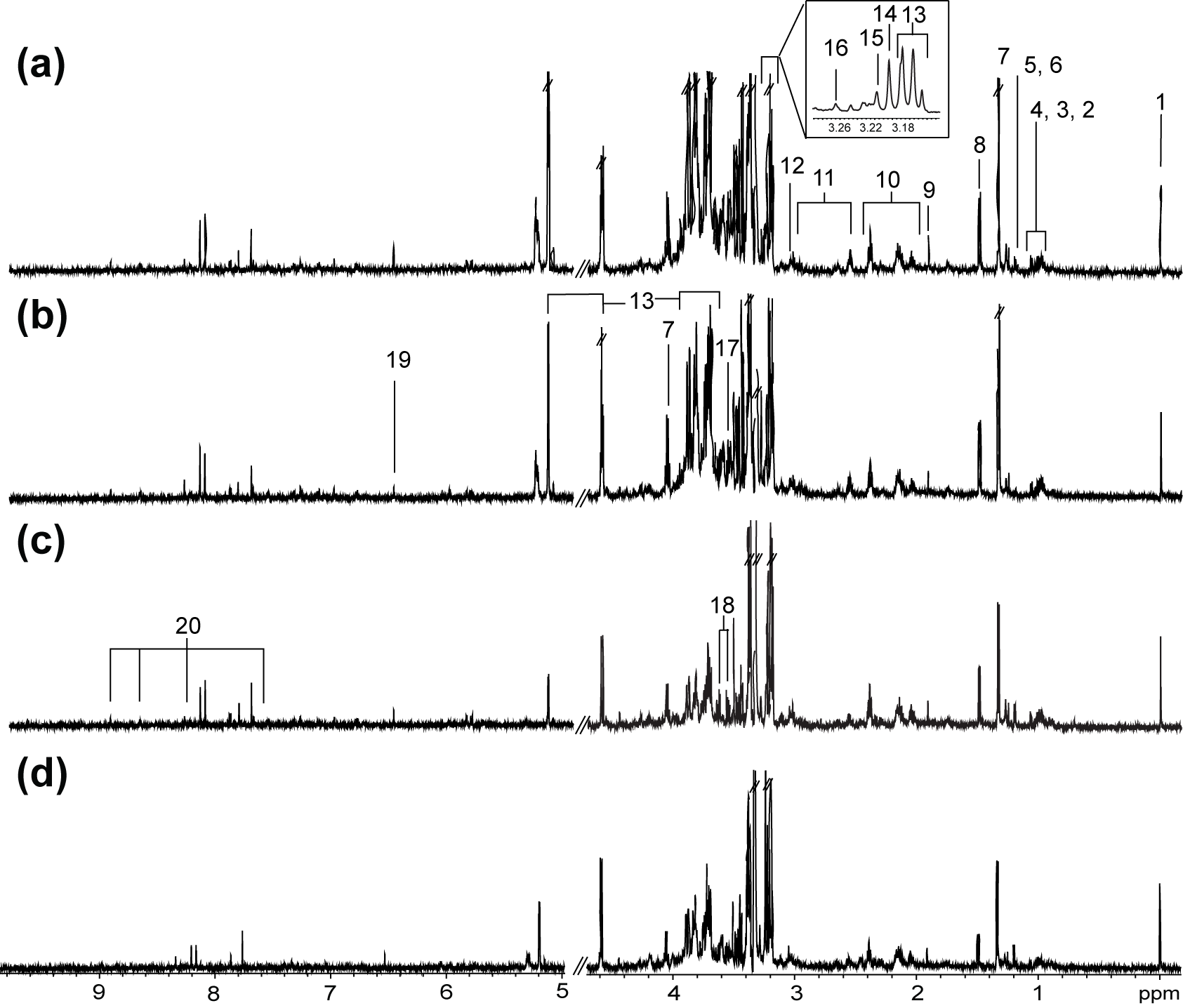

Supplement: S2 Fig — Representative 600MHz 1H – NMR spectra of liver extract of control mice for day 4 (a) control mice for day 7 (b), O. tsutsugamushi -infected mice for day 4 (c), O. tsutsugamushi -infected mice for day 7 (d). Key, 1. TSP, 2. Isoleucine, 3. Leucine, 4. Valine, 5. Isopropanol, 6. 3-Hydroxybutyrate, 7. Lactate, 8. Alanine, 9. Acetate, 10. Glutamate, 11. Glutathione 12. Creatine, 13. Glucose, 14. Choline, 15. O-Phosphocholine, 16. Betaine, 17. Glycine, 18. Glycerol, 19. Fumarate, 20. Nicotinurate. (TIF) [file pntd.0003427.s002.tif]

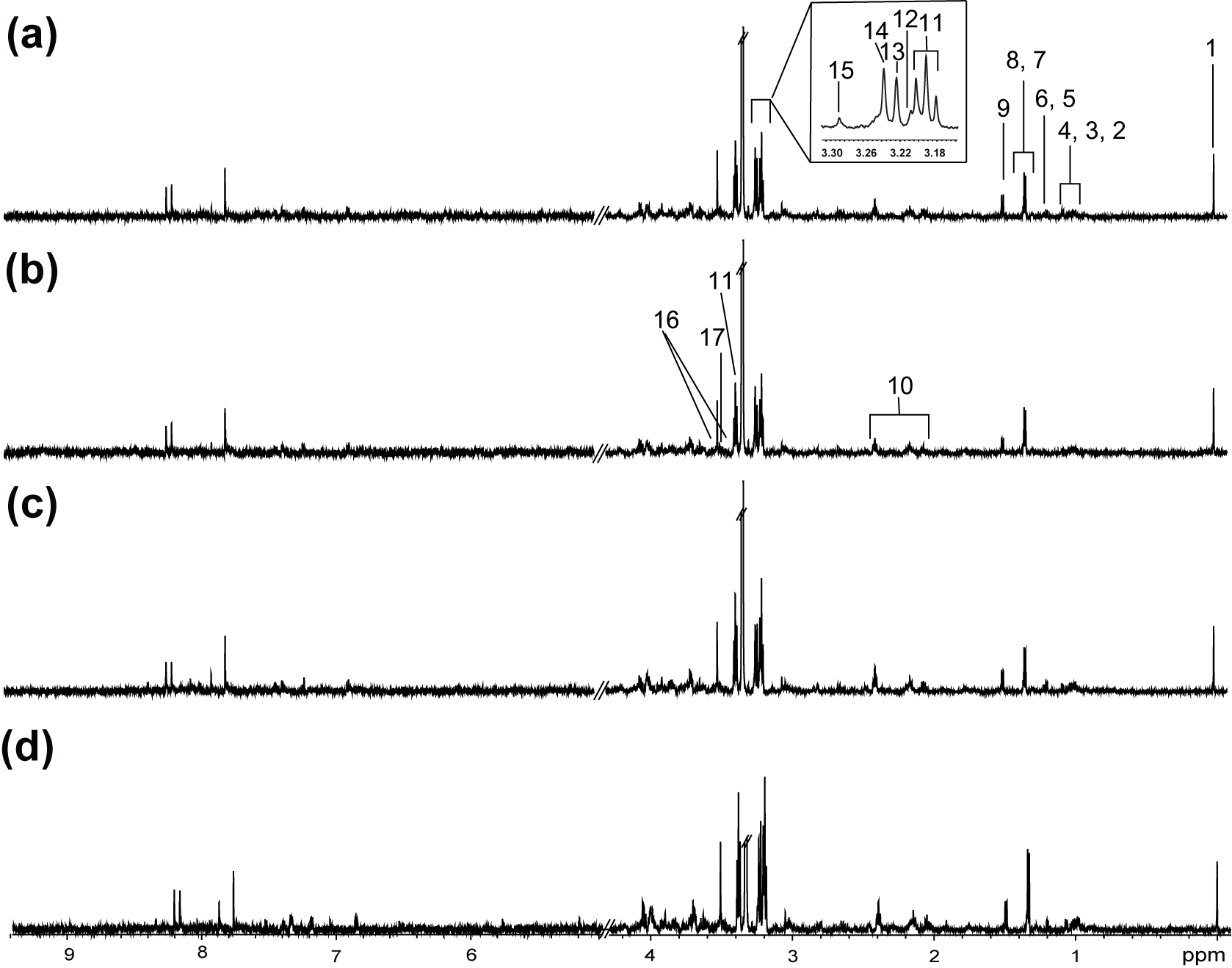

Supplement: S3 Fig — Representative 600 MHz 1H – NMR spectra of spleen extract of control mice for day 4 (a) control mice for day 7 (b), O. tsutsugamushi -infected mice for day 4 (c), O. tsutsugamushi -infected mice for day 7 (d). Key, 1. TSP, 2. Isoleucine, 3. Leucine, 4. Valine, 5. Isopropanol, 6. 3-Hydroxybutyrate, 7. Lactate 8. Threonine, 9. Alanine, 10. Glutamate, 11. Taurine, 12.O-Phosphoethanolamine 13. Choline, 14. O-Phosphocholine, 15 Betaine, 16. Glycine, 17. Myo-inositol. (TIF) [file pntd.0003427.s003.tif]

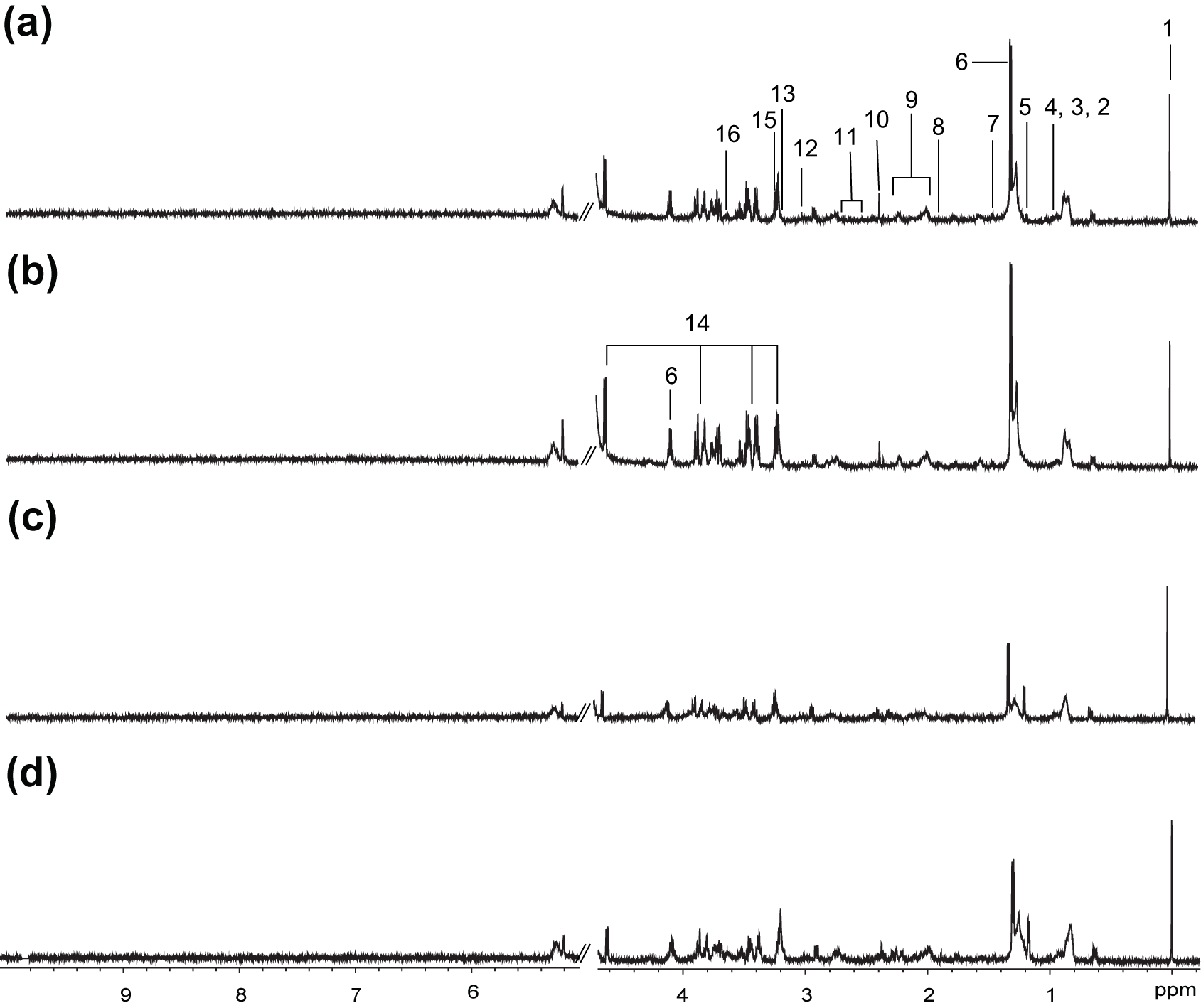

Supplement: S4 Fig — Representative 600 MHz 1H – NMR spectra of serum of control mice for day 4 (a) control mice for day 7 (b), O. tsutsugamushi -infected mice for day 4 (c), O. tsutsugamushi -infected mice for day 7 (d). Key, 1. DSS, 2. Isoleucine 3. Leucine, 4. Valine, 5. 3-Hydroxybutyrate, 6. Lactate, 7. Alanine, 8. Acetate, 9. Glutamate, 10. Pyruvate, 11. Citrate, 12. Creatine, 13. Choline, 14. Glucose, 15. Betaine, 16. Glycerol. (TIF) [file pntd.0003427.s004.tif]

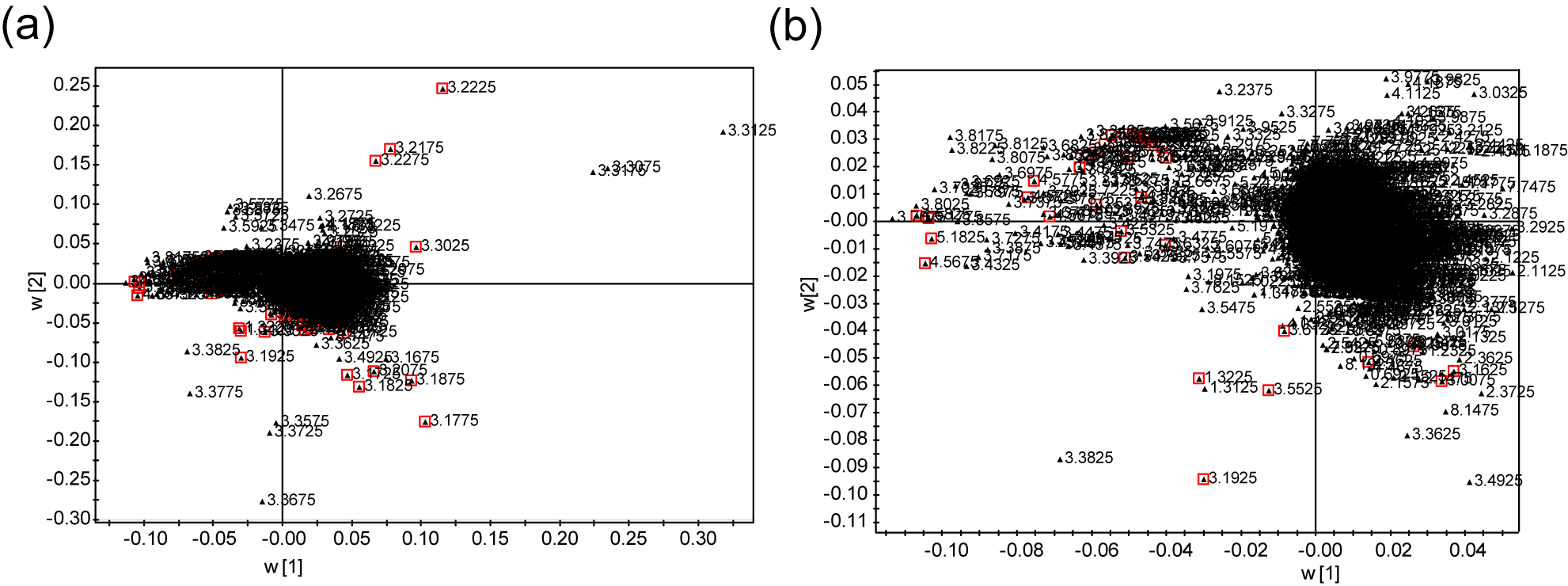

Supplement: S5 Fig — Loading plots of OPLS-DA model derived from 1H NMR spectra of liver tissue: (a), full scale and (b), expanded scale. Variables having high values of weight in OPLS-DA model were marked with red square. (TIF) [file pntd.0003427.s005.tif]

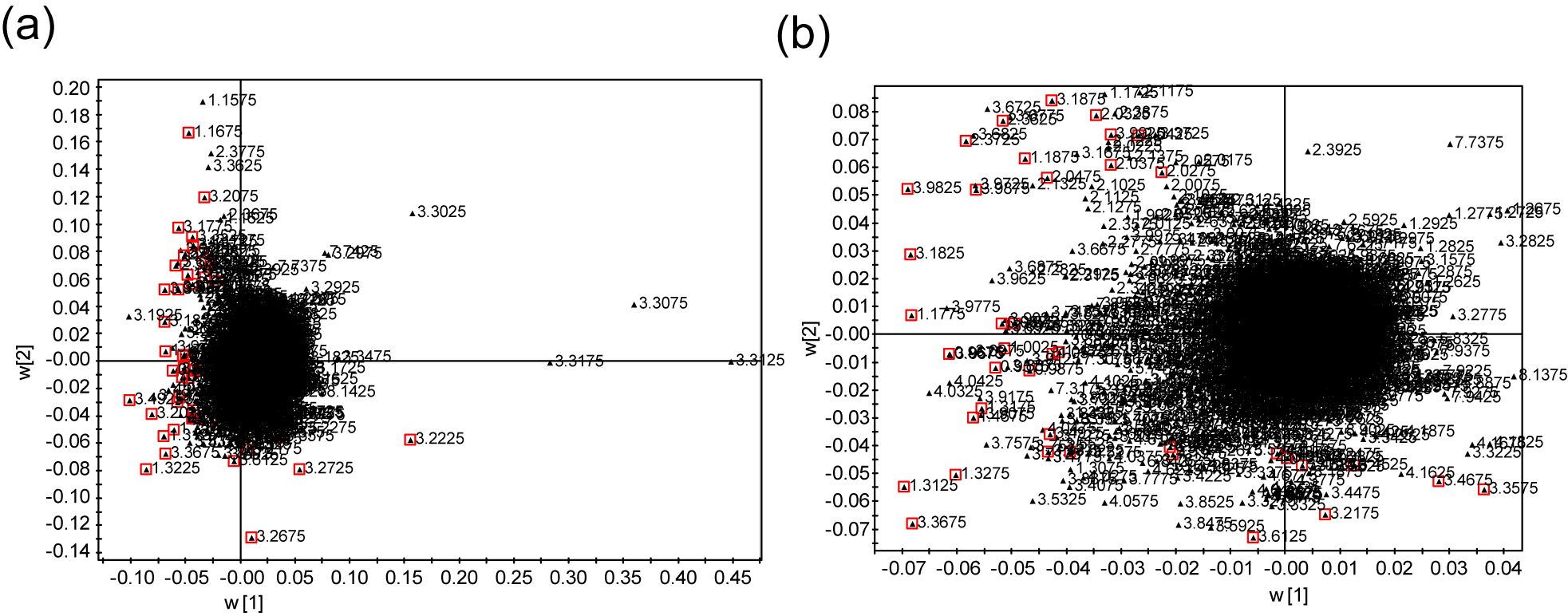

Supplement: S6 Fig — Loading plots of OPLS-DA model derived from 1H NMR spectra of spleen tissue: (a), full scale and (b), expanded scale. Variables having high values of weight in OPLS-DA model were marked with red square. (TIF) [file pntd.0003427.s006.tif]

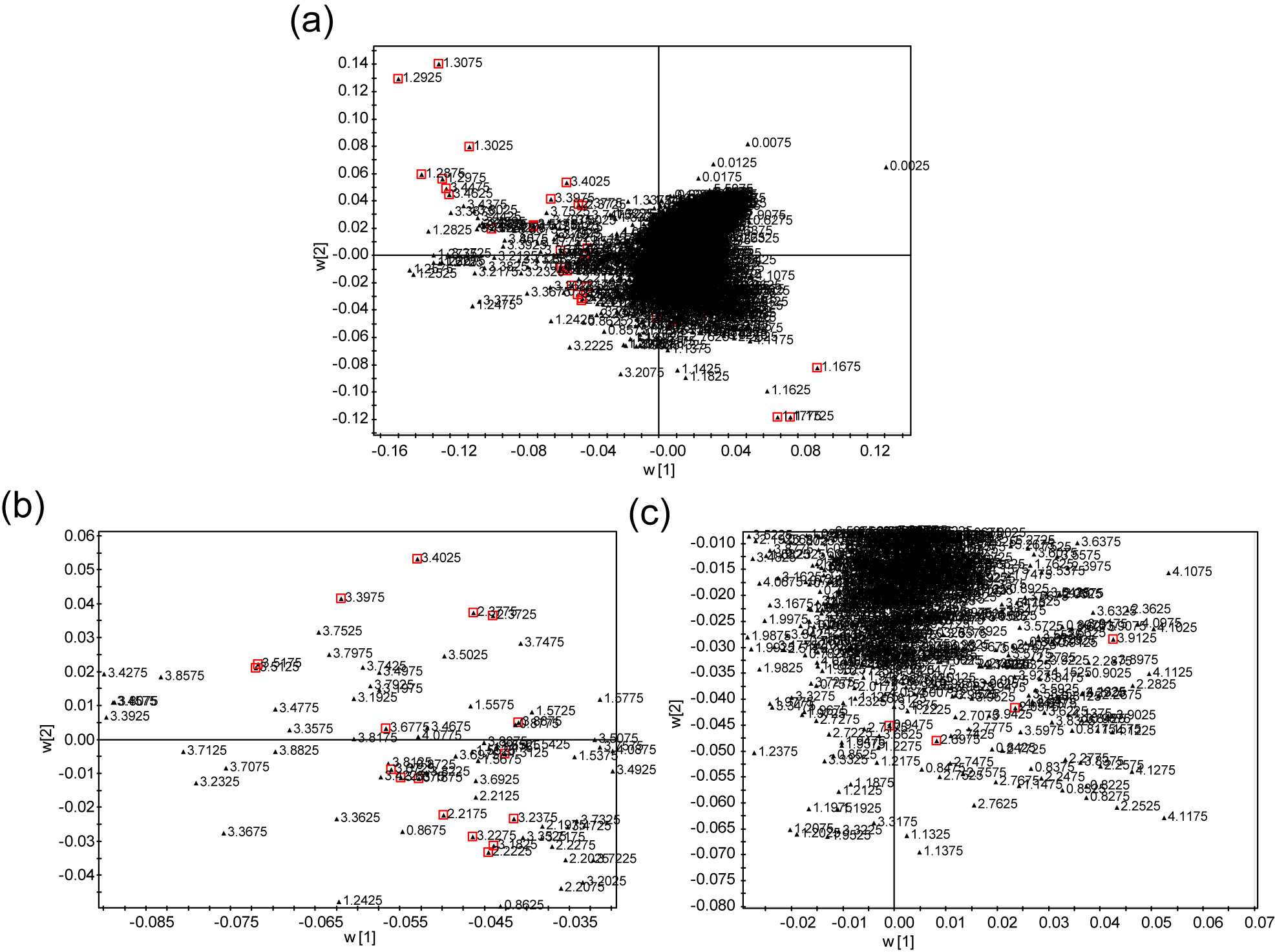

Supplement: S7 Fig — Loading plots of OPLS-DA model derived from 1H NMR spectra of serum: (a), full scale and (b, c), expanded scale. Variables having high values of weight in OPLS-DA model were marked with red square. (TIF) [file pntd.0003427.s007.tif]
