# Supplementary material for: Metabolic Responses to Orientia tsutsugamushi Infection in a Mouse Model
Source: PLoS Negl Trop Dis. 2015 Jan 8;9(1):e3427. doi: 10.1371/journal.pntd.0003427 (PMC4287389; doi:10.1371/journal.pntd.0003427)
Supplement: S1 Table — Analysis of phosphatidylcholin and phosphatidylethanolamine of spleen. (DOCX) [file pntd.0003427.s008.docx]

**Table S1**. **Anlaysis of phosphatidylcholin and phosphatidylethanolamine of spleen.**

| Fatty acids | | class | con_4day | | | karp_4day | | |  | con_7day | | | karp_7day | | |  |
| --- | --- | --- | --- | --- | --- | --- | --- | --- | --- | --- | --- | --- | --- | --- | --- | --- |
| 36:1 | 18:0/18:1 | PC | 49822 | ± | 5429 | 53071 | ± | 3847 |  | 52867 | ± | 5756 | 70182 | ± | 13364 | ***** |
|  |  | PE | 7290 | ± | 359 | 7744 | ± | 655 |  | 8309 | ± | 1113 | 10460 | ± | 1250 | ***** |
|  |  | PC/PE | 6.8 | ± | 0.7 | 6.9 | ± | 0.2 |  | 6.4 | ± | 0.5 | 6.7 | ± | 0.8 |  |
| 36:2 | 18:0/18:2 | PC | 113078 | ± | 22628 | 132254 | ± | 16791 |  | 121341 | ± | 15894 | 191711 | ± | 45943 | ***** |
|  |  | PE | 13391 | ± | 1709 | 15362 | ± | 948 |  | 16366 | ± | 2538 | 22089 | ± | 2721 | ***** |
|  |  | PC/PE | 8.4 | ± | 0.8 | 8.6 | ± | 0.8 |  | 7.4 | ± | 0.3 | 8.6 | ± | 1.1 |  |
| 36:3 | 18:1/18:2 | PC | 45887 | ± | 12933 | 63619 | ± | 10109 | ***** | 52497 | ± | 8424 | 95809 | ± | 32824 | ***** |
|  |  | PE | 3570 | ± | 711 | 4514 | ± | 217 | ***** | 4867 | ± | 1229 | 6062 | ± | 705 |  |
|  |  | PC/PE | 12.8 | ± | 2.0 | 14.1 | ± | 2.5 |  | 11.0 | ± | 1.0 | 15.5 | ± | 3.8 |  |
| 36:4 | 18:2/18:2 | PC | 10785 | ± | 4711 | 14575 | ± | 2212 |  | 11817 | ± | 2628 | 23973 | ± | 7312 | ***** |
|  |  | PE | 672 | ± | 163 | 929 | ± | 91 | ***** | 790 | ± | 123 | 1045 | ± | 152 |  |
|  |  | PC/PE | 15.7 | ± | 3.5 | 15.7 | ± | 2.4 |  | 14.9 | ± | 1.6 | 22.5 | ± | 4.1 | ***** |
|  | 16:0/20:4 | PC | 294550 | ± | 47505 | 289511 | ± | 18135 |  | 336428 | ± | 26068 | 330000 | ± | 68126 |  |
|  |  | PE | 25094 | ± | 1695 | 26787 | ± | 1913 |  | 31311 | ± | 3585 | 23170 | ± | 4023 | ***** |
|  |  | PC/PE | 11.8 | ± | 2.2 | 10.8 | ± | 1.0 |  | 10.8 | ± | 0.6 | 14.8 | ± | 5.2 | ***** |
| 36:5 | 16:0/20:5 | PC | 4812 | ± | 708 | 4213 | ± | 1330 |  | 7140 | ± | 4171 | 5044 | ± | 1600 |  |
|  |  | PE | 1832 | ± | 270 | 2209 | ± | 799 |  | 2224 | ± | 709 | 1403 | ± | 538 |  |
|  |  | PC/PE | 2.7 | ± | 0.6 | 2.0 | ± | 0.3 |  | 3.1 | ± | 0.9 | 3.8 | ± | 1.5 |  |
|  | 16:1/20:4 | PC | 9026 | ± | 824 | 9423 | ± | 817 |  | 10494 | ± | 1260 | 9254 | ± | 633 | ***** |
|  |  | PE | 625 | ± | 141 | 643 | ± | 203 |  | 787 | ± | 127 | 482 | ± | 175 |  |
|  |  | PC/PE | 15.0 | ± | 3.4 | 15.6 | ± | 3.7 |  | 13.4 | ± | 1.0 | 20.7 | ± | 5.9 |  |
| 38:3 | 18:0/20:3 | PC | 8911 | ± | 1310 | 7989 | ± | 1061 |  | 9349 | ± | 561 | 9748 | ± | 1628 |  |
|  |  | PE | 3464 | ± | 160 | 3436 | ± | 245 |  | 3917 | ± | 537 | 3709 | ± | 318 |  |
|  |  | PC/PE | 2.6 | ± | 0.3 | 2.3 | ± | 0.2 |  | 2.4 | ± | 0.2 | 2.6 | ± | 0.3 |  |
| 38:4 | 16:0/22:4 | PC | 47769 | ± | 7290 | 53356 | ± | 5823 |  | 47468 | ± | 9460 | 47579 | ± | 10443 |  |
|  |  | PE | 8426 | ± | 1209 | 10416 | ± | 1122 | ***** | 9343 | ± | 1413 | 7157 | ± | 1503 |  |
|  |  | PC/PE | 5.7 | ± | 1.1 | 5.2 | ± | 0.9 |  | 5.1 | ± | 0.6 | 7.0 | ± | 2.3 |  |
|  | 18:0/20:4 | PC | 151295 | ± | 23447 | 153098 | ± | 14690 |  | 165802 | ± | 17986 | 181733 | ± | 43095 |  |
|  |  | PE | 81427 | ± | 3839 | 86676 | ± | 4782 |  | 96815 | ± | 11631 | 90498 | ± | 6584 |  |
|  |  | PC/PE | 1.9 | ± | 0.2 | 1.8 | ± | 0.2 |  | 1.7 | ± | 0.1 | 2.0 | ± | 0.5 |  |
| 38:5 | 18:1/20:4 | PC | 87797 | ± | 7620 | 90765 | ± | 1866 |  | 97456 | ± | 12891 | 101866 | ± | 18757 |  |
|  |  | PE | 19769 | ± | 3363 | 22242 | ± | 2891 |  | 24695 | ± | 3604 | 19473 | ± | 1654 | ***** |
|  |  | PC/PE | 4.5 | ± | 0.8 | 4.1 | ± | 0.6 |  | 4.0 | ± | 0.4 | 5.3 | ± | 1.1 | ***** |
| 38:6 | 18:2/20:4 | PC | 20649 | ± | 2548 | 23153 | ± | 959 |  | 24394 | ± | 1740 | 29422 | ± | 6040 |  |
|  |  | PE | 13840 | ± | 3544 | 22493 | ± | 4317 | ***** | 16021 | ± | 3442 | 14864 | ± | 3271 |  |
|  |  | PC/PE | 1.6 | ± | 0.4 | 1.1 | ± | 0.2 | ***** | 1.6 | ± | 0.3 | 2.1 | ± | 0.8 |  |
| 40:7 | 18:1/22:6 | PC | 22122 | ± | 3829 | 29522 | ± | 2499 | ***** | 23366 | ± | 2309 | 32744 | ± | 6478 | ***** |
|  |  | PE | 18874 | ± | 2471 | 22403 | ± | 1899 | ***** | 22671 | ± | 2976 | 20123 | ± | 1422 |  |
|  |  | PC/PE | 1.2 | ± | 0.2 | 1.3 | ± | 0.2 |  | 1.0 | ± | 0.1 | 1.6 | ± | 0.2 | ***** |
| ***** | p<0.05 |  |  |  |  |  |  |  |  |  |  |  |  |  |  |  |
