# Supplementary material for: Metabolic Responses to Orientia tsutsugamushi Infection in a Mouse Model
Source: PLoS Negl Trop Dis. 2015 Jan 8;9(1):e3427. doi: 10.1371/journal.pntd.0003427 (PMC4287389; doi:10.1371/journal.pntd.0003427)
Supplement: S2 Table — Analysis of fatty acids of spleen. (DOCX) [file pntd.0003427.s009.docx]

**Table S2**. **Anlaysis of fatty acids of spleen.**

|  | Day 4 | | | | | | | | |  | Day7 | | | | | |  |
| --- | --- | --- | --- | --- | --- | --- | --- | --- | --- | --- | --- | --- | --- | --- | --- | --- | --- |
| class | Control | | | | | Karp | | | |  | Control | | | Karp | | |  |
| **Saturated fatty acid** | | |  |  | |  | |  |  |  |  |  |  |  |  |  |  |
| FFA 16:0 | 273543 | ± | | 61768 | | 241445 | | ± | 32571 |  | 323118 | ± | 61358 | 384140 | ± | 186733 |  |
| FFA 18:0 | 244752 | ± | | 65036 | | 220749 | | ± | 38562 |  | 271398 | ± | 87841 | 349799 | ± | 179728 |  |
| **Monounsaturated fatty acid** | | | | |  |  | |  |  |  |  |  |  |  |  |  |  |
| FFA 16:1 | 8517 | ± | | 4036 | | 9832 | | ± | 1564 |  | 33526 | ± | 37362 | 22600 | ± | 8031 |  |
| FFA 18:1 | 92463 | ± | | 39937 | | 132413 | | ± | 35927 |  | 139644 | ± | 67064 | 311419 | ± | 178233 |  |
| FFA 20:1 | 4456 | ± | | 1688 | | 6897 | | ± | 2272 |  | 4651 | ± | 1072 | 18163 | ± | 14210 |  |
| **Polyunsaturated fatty acid** | | | | |  |  | |  |  |  |  |  |  |  |  |  |  |
| FFA 18:2 | 49203 | ± | | 29365 | | 69348 | | ± | 21101 |  | 51167 | ± | 23258 | 171900 | ± | 101906 | ***** |
| FFA 18:3 | 1749 | ± | | 1228 | | 1572 | | ± | 329 |  | 1657 | ± | 1316 | 3527 | ± | 1840 |  |
| FFA 20:2 | 4073 | ± | | 2516 | | 7820 | | ± | 3047 |  | 3620 | ± | 776 | 21136 | ± | 15302 |  |
| FFA 20:3 | 2833 | ± | | 2179 | | 5636 | | ± | 2255 |  | 2732 | ± | 713 | 17144 | ± | 14283 |  |
| FFA 20:4 | 16583 | ± | | 12154 | | 25994 | | ± | 8250 |  | 21817 | ± | 7040 | 105292 | ± | 105149 |  |
| FFA 20:5 | 279 | ± | | 168 | | 367 | | ± | 87 |  | 465 | ± | 359 | 1809 | ± | 1698 |  |
| **Estimated delta 6 desaturase activity** | | | | | | |  |  |  |  |  |  |  |  |  |  |  |
| 20:4/18:2 | 0.33 | ± | | 0.08 | | 0.38 | | ± | 0.05 |  | 0.45 | ± | 0.10 | 0.48 | ± | 0.30 |  |
